# Neurodevelopmental Changes in Excitatory Synaptic Structure and Function in the Cerebral Cortex of Sanfilippo Syndrome IIIA Mice

**DOI:** 10.1038/srep46576

**Published:** 2017-04-18

**Authors:** Chrissa A. Dwyer, Samantha L. Scudder, Ying Lin, Lara E. Dozier, Dustin Phan, Nicola J. Allen, Gentry N. Patrick, Jeffrey D. Esko

**Affiliations:** 1 Department of Cellular and Molecular Medicine, Glycobiology Research and Training Center, University of California, San Diego, La Jolla, CA, 92093-0687, USA; 2 Section of Neurobiology, Division of Biological Sciences, University of California, San Diego, La Jolla, CA 92093-0347, USA; 3 Salk Institute for Biological Studies, Molecular Neurobiology Laboratory, La Jolla, CA 92037, USA

## Abstract

Sanfilippo syndrome, MPS IIIA-D, results from deficits in lysosomal enzymes that specifically degrade heparan sulfate, a sulfated glycosaminoglycan. The accumulation of heparan sulfate results in neurological symptoms, culminating in extensive neurodegeneration and early death. To study the impact of storage in postnatal neurodevelopment, we examined murine models of MPS IIIA, which lack the enzyme sulfamidase. We show that changes occur in excitatory postsynaptic structure and function in the somatosensory cortex prior to signs of neurodegeneration. These changes coincide with accumulation of heparan sulfate with characteristic non-reducing ends, which is present at birth in the mutant mice. Accumulation of heparan sulfate was also detected in primary cultures of cortical neural cells, especially astrocytes. Accumulation of heparan sulfate in cultured astrocytes corresponded with augmented extracellular heparan sulfate and glypican 4 levels. Heparan sulfate from the cerebral cortex of MPS IIIA mice showed enhanced ability to increase glutamate AMPA receptor subunits at the cell surface of wild type neurons. These data support the idea that abnormalities in heparan sulfate content and distribution contribute to alterations in postsynaptic function. Our findings identify a disease-induced developmental phenotype that temporally overlaps with the onset of behavioral changes in a mouse model of MPS IIIA.

All cell types in vertebrates and invertebrates, including cells in the central nervous system (CNS), produce plasma membrane and extracellular matrix heparan sulfate proteoglycans (HSPGs). HSPGs consist of a protein core with one or more covalently attached heparan sulfate (HS) chains. HS is comprised of alternating glucosamine and uronic acids (d-glucuronic and l-iduronic) variously modified by sulfate groups^1^. Studies of animal models deficient in HS biosynthesis show that HS has a role in nearly every developmental process in the brain, including neural progenitor cell proliferation and differentiation^2^,^3^,^4^, axon guidance^5^,^6^ and synapse formation^7^,^8^. HS in the adult brain also plays a role in maintaining synaptic function^9^. These processes depend on the binding of numerous growth factors, receptors and guidance proteins to sulfated domains within the chains^1^.

HS turnover occurs through several mechanisms, including proteolytic shedding of proteoglycans from the cell surface, limited endolytic cleavage of the chains by heparanase, and endocytosis, which culminates in lysosomal degradation. Degradation of HS in the lysosome occurs in a sequential manner beginning at the non-reducing end of the chain. Thus, inactivating mutations in any of the degradative enzymes arrests the process, causing accumulation of HS fragments in the lysosome. In humans and animal models, storage results in mucopolysaccharidosis (MPS), a lysosomal storage disorder^10^. Sanfilippo syndromes (MPS IIIA-D) arise from deficits in enzymes that hydrolyze or modify the non-reducing terminal glucosamine unit. MPS IIIA (OMIM 252900) is a homozygous recessive, congenital disorder caused by the lack of the enzyme sulfamidase (SGSH), which removes the *N*-sulfate group from terminal *N*-sulfoglucosamine units. Failure to degrade the chains and/or accumulation of chains with terminal *N*-sulfoglucosamine units results in cell death and neurodegeneration. The syndrome also manifests behavioral changes during childhood^11^,^12^. MPS IIIB (OMIM 252920), MPS IIIC (OMIM 252930) and MPS IIID (OMIM 252940), caused by deficiencies in other enzymes involved in lysosomal processing of the non-reducing terminal glucosamine residue in HS, have similar clinical consequences. Recently, mutation of a fifth enzyme involved in processing of terminal glucosamine residues (3-sulfoglucosamine sulfatase) was shown to result in lysosomal storage and neuropathology in mice^13^.

Sanfilippo syndrome affects the central nervous system and is considered a childhood neurodegenerative disorder. MPS III children present typically with developmental delay that can be accompanied by severe hyperactivity, autistic-like social behaviors and insomnia. In animal models of MPS III, the appearance of abnormal neurological behaviors precedes neuronal cell loss and nervous system atrophy^14^,^15^. This observation suggests that changes in neuronal circuit function may give rise to neurological dysfunction at disease onset. Here we show that HS accumulation occurs at birth in the developing MPS IIIA brain and impacts neuronal function at early time points in disease pathogenesis. The results demonstrate that MPS IIIA mice exhibit deficits in synaptic function in the developing somatosensory cortex, which may explain early behavioral abnormalities in MPS III patients.

## Results

### Alterations in excitatory synaptic structure and function occurs in the developing brain of Sgsh mutant mice

**Figure 1:**
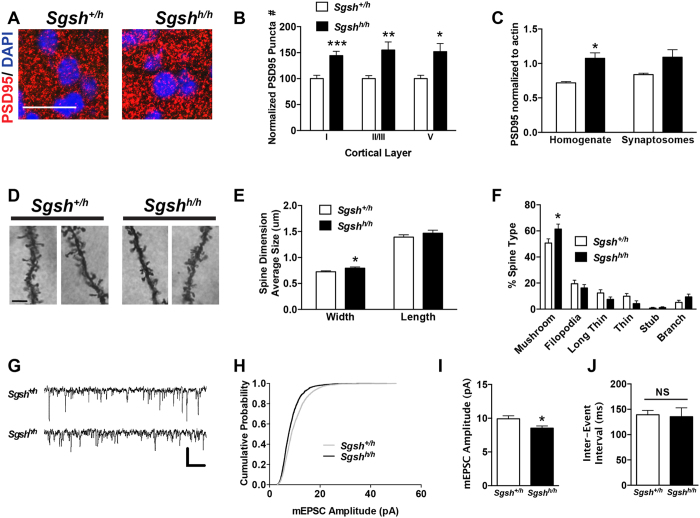
Excitatory postsynaptic structure and function of layer II/III pyramidal neurons from the primary somatosensory cortex of postnatal MPS IIIA mice is abnormal. (**A**) Representative confocal images show an increase in PSD-95 puncta (red) in layer II/III of the primary somatosensory cortex in 21d (day-old) hypomorphic MPS IIIA (*Sgsh*^*h/h*^) mice. Scale, 50 μm. Nuclei are stained with DAPI (blue). (**B**) Quantification of PSD-95 puncta in cortical layers I, II/III, and V of the primary somatosensory cortex of 21d unaffected *Sgsh*^+/*h*^ and *Sgsh*^*h/h*^ mice. Mean ± SEM. N = 5–6 animals per genotype. (**C**) Western blot for PSD-95 levels in cortical homogenate and synaptosome preparations from 21d unaffected and hypomorphic MPS IIIA mice. Band pixel intensities were measured in ImageJ and normalized to β-actin. Mean ± SEM. N = 3 animals per genotype, spanning 2 litters. (**D**) Representative maximum intensity projection z-stacks of dendritic segments from pyramidal neurons in layer II/III of the somatosensory cortex of 21d unaffected and hypomorphic MPS IIIA mice. (**E**) Quantification of dendritic spine head width and shaft length of pyramidal neurons in layer II/III of the somatosensory cortex of 21d unaffected and hypomorphic MPS IIIA mice. Mean ± SEM. N = 3 animals per genotype. (**F**) Quantification of dendritic spine type. Mean ± SEM. (**G**) Representative mEPSC recordings from pyramidal neurons in layer II/III of the somatosensory cortex of 21–22d unaffected and hypomorphic MPS IIIA mice. Scale bar depicts 20 pA, 200 msec. (**H**) Cumulative probability distributions of all mEPSC event amplitudes recorded from pyramidal neurons in layer II/III of the somatosensory cortex of 21–22d unaffected and hypomorphic MPS IIIA (mice, depicting reduced amplitude in mutant animals. N = 1883, 2362 events. p < 0.001, Kolmogorov-Smirnov test. (**I)** Mean mEPSC amplitude. Mean ± SEM. (**J**) Mean mEPSC inter-event interval. N = 5 mice per genotype, 16–19 cells total. *p* = 0.015, Student’s t test.

To determine whether synaptic development was altered in MPS IIIA mice, we used a naturally occurring hypomorphic mutant (*Sgsh*^*h/h*^)^16^,^17^. This line contains a missense mutation in the sulfamidase gene, *Sgsh*, reducing enzyme activity by >95%. *Sgsh*^*h/h*^ mice exhibit hyperactivity and signs of reduced anxiety as early as 3 weeks of age, which is exaggerated by repeated behavioral testing^18^,^19^. Early behavioral abnormalities in human patients and mouse models are consistent with manifestation of disease pathology in the somatosensory cortex, a region of the cerebral cortex involved in the integration of sensory input. Formation and refinement of the neural circuitry in this region of the brain occurs from postnatal day 7 to 28 in mice. Staining of sections of postnatal day 21 somatosensory cortex with the excitatory postsynaptic marker postsynaptic density 95 (PSD-95) showed enhanced puncta number in cortical layers I, II/III and V in *Sgsh*^*h/h*^ mice compared to heterozygous mice (*Sgsh*^+/h^), which do not store HS or present any disease symptoms (Fig. 1A,B; Fig. S1A,B). Western blot analysis on whole tissue homogenates and synaptosomes prepared from the cerebral cortex confirmed the accumulation of PSD-95 in *Sgsh*^*h/h*^ mice (Fig. 1C). The increase in PSD-95 was not observed in 14-day old mice (Fig. S1C). In the healthy brain, the postsynaptic apparatus containing PSD-95 localizes to the head of membranous protrusions called dendritic spines. Golgi silver-impregnation of layer II/III pyramidal neurons in the primary somatosensory cortex of *Sgsh*^*h/h*^ mice at day 21 showed that the number of dendritic spines was not altered (*Sgsh*^+/*h*^ 0.84 ± 0.12, *Sgsh*^*h/h*^ 0.79 ± 0.11 protrusions/μm, *p* > 0.05), but a modest increase in spine head width (Fig. 1D,E) and the percentage of mushroom-shaped spines was observed (Fig. 1F). These findings suggest that synapse architecture is altered by the end of postnatal neurodevelopment in *Sgsh*^h/h^ mice.

Synapse strength closely correlates with dendritic spine morphology (reviewed in ref. 20). To determine whether excitatory synaptic function was altered, electrophysiological recordings were performed in layer II/III pyramidal neurons in the primary somatosensory cortex of acutely isolated brain slices from 21-day old *Sgsh*^*h/h*^ mice (Fig. 1G). Evaluation of total miniature excitatory postsynaptic current (mEPSC) events revealed fewer large events in the mutant (Fig. 1H). Average mEPSCs amplitude was also significantly reduced in the mutant (Fig. 1I), showing that excitatory postsynaptic strength is reduced in *Sgsh*^*h/h*^ mice. No change in mEPSC inter-event interval (Fig. 1J) was observed, consistent with the absence of change in the number of dendritic spines. These findings reflect reduced glutamatergic excitatory synaptic strength in layer II/III pyramidal cortical neurons without alterations in synapse number or presynaptic neurotransmitter vesicle release. Evaluation of α-amino-3-hydroxy-5-methyl-4-isoxazolepropionic acid (AMPA) receptor subunit levels, GluA1 and GluA2, showed no change in *Sgsh*^*h/h*^ mice by Western blotting (Fig. S1D,E) and surface expression by biotinylation (Fig. S1F,G). Thus, reduced glutamatergic synaptic strength does not reflect a loss of GluA1 or GluA2 receptors.

To determine whether changes in synaptic function occurred in other brain regions, we also assessed a form of short-term synaptic plasticity in the hippocampus as measured by excitatory synaptic basal transmission and paired pulse facilitation. Basal synaptic transmission in the Schaffer collateral pathway in acute hippocampal slices had normal field excitatory postsynaptic potentials (fEPSPs) in response to increasing stimulation intensities (Fig. S2A). Paired-pulse facilitation in these hippocampi was intact, although a small reduction in facilitation with 50 msec separation of pulses was observed (Fig. S2B). Thus, MPS IIIA mice exhibit specific deficits in excitatory postsynaptic structure and function in the developing postnatal primary somatosensory cortex.

### Lysosomal expansion, but not astrocytosis and ganglioside accumulation, are present in the developing postnatal somatosensory cortex

**Figure 2:**
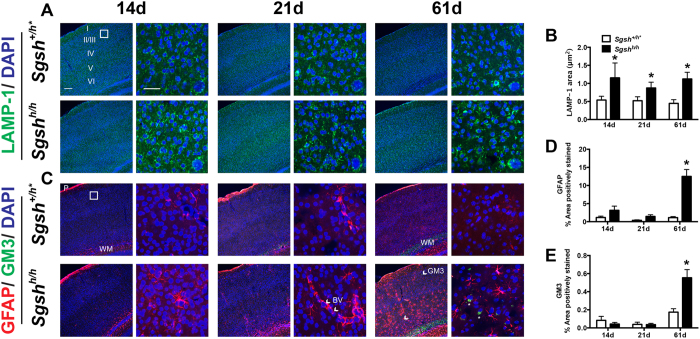
Lysosomal expansion but not astrocytosis and ganglioside accumulation are present in the developing postnatal primary somatosensory cortex. (**A**) Representative confocal images from unaffected (*Sgsh*^+/h*^) and hypomorphic MPS IIIA (*Sgsh*^*h/h*^) mouse primary somatosensory cortex stained with LAMP-1 (green). Left panels show low magnification images, scale bar 100 μm. Right panels show high magnification of layer II/III (boxed region), scale bar 50 μm. Insets in lower right corner show single cells. Nuclei were stained with DAPI (blue). (**B**) Quantification of LAMP-1 stained vesicle area from high magnification images. (**C**) Representative confocal images stained with a marker for reactive cortical astrocytes, GFAP (red) and ganglioside GM3 (green), as described in (**A**). Arrows in low magnification images point to cells with intracellular staining of GM3, denoted by asterisks in high magnification images. *P*, pial basement membrane; *WM*, white matter; *BV*, blood vessel. (**D**) Quantification of GFAP immunoreactivity in the parenchyma of the somatosensory cortex. (**E**) Quantification of GM3 immunoreactivity in the parenchyma of the somatosensory cortex. N = 3 animals per genotype per age, 2 sections per animal, 2 regions of interest per section were quantified. All brains were collected, stained, and analyzed at the same time. Findings were confirmed on a separate cohort of N = 3 animals per genotype per age. Mean ± SD.

To determine whether changes in postsynaptic structure and function might be due to lysosomal expansion in mutant animals, we analyzed the lysosomal marker, LAMP-1, in 14, 21 and 61 day old mice. Enhanced LAMP-1 immunostaining was detected throughout the cortical layers of the primary somatosensory cortex at all ages in *Sgsh*^*h/h*^ mice relative to littermate *Sgsh*^+/*h*^ controls (Fig. 2A,B). We also evaluated astrogliosis and secondary accumulation of GM3 ganglioside, traits associated with neurodegenerative stages of disease progression in the aged MPS IIIA brain. Staining tissue sections with antibodies against glial fibrillary protein (GFAP) showed reactive astrocytes along the pial basement membrane (P), a subset of blood vessels (BV) and white matter (WM) tracks in control mice (Fig. 2C). Staining in the vicinity of the blood vessels and white matter tracts was elevated in *Sgsh*^h/h^ mice (Fig. 2C), culminating at 61 days of age in widespread distribution of GFAP-positive astrocytes throughout the somatosensory cortex (Fig. 2C,D). GM3 ganglioside also accumulated in cortical layers II/III and V of the somatosensory cortex of 61-day-old adult mutant mice (Fig. 2C,E), but not in younger or unaffected mice. Thus, lysosomal expansion and post-synaptic dysfunction precedes widespread astrocytosis and secondary storage of GM3 ganglioside.

### Heparan sulfate storage occurs at birth and increases coincident with postnatal brain growth

**Figure 3:**
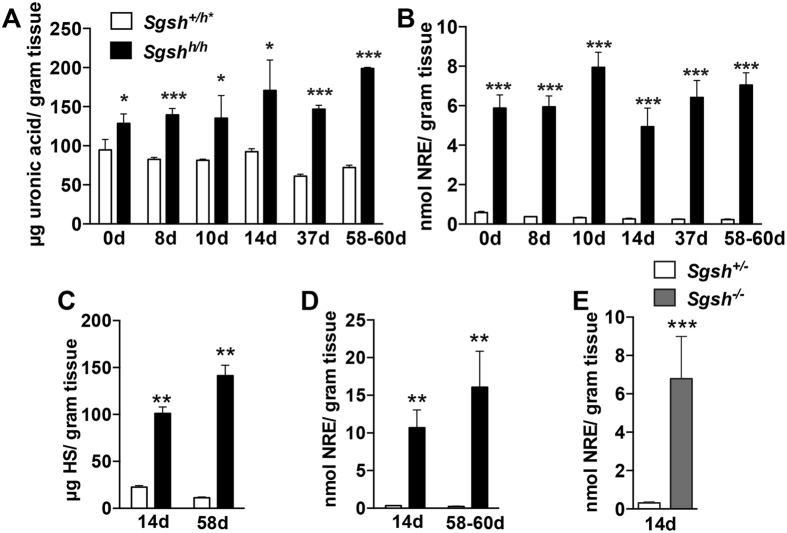
Accumulation of undegraded heparan sulfate occurs in the developing postnatal MPS IIIA mouse brain and cerebral cortex. (**A**) Whole brain glycosaminoglycan uronic acid levels by carbazole assay. (**B**) Whole brain NRE levels (*N*-sulfoglucosamine) by LC/MS. (**A**,**B**) values are normalized to brain wet weight, which is presented in Fig. S3A. (**C**) Cerebral cortex heparan sulfate levels measured by LC/MS. (**D**) Cerebral cortex NRE levels by LC/MS. (**E**) *Sgsh* null (*Sgsh*^−/−^) cerebral cortex NRE levels by LC/MS. Values normalized to wet tissue mass. Unaffected wild type and heterozygous (*Sgsh*^+/h*^ open bars) and hypomorphic MPS IIIA (*Sgsh*^*h/h*^ closed bars), unless otherwise specified. Day of birth = 0 day. N = 3 animals per genotype per age. Transcardial perfusion with PBS was performed on all animals to eliminate contamination from residual blood in tissue. In all cases graphs show mean ± SD.

Accumulation of LAMP-1 stained lysosomes is consistent with storage of HS fragments. HS that accumulates in MPS IIIA has diagnostic terminal non-reducing end (NRE) glycan structures that serve as biomarkers of the disease^21^. Digestion of the chains with bacterial lyases releases these NRE glycans, which can be readily quantified by liquid chromatography/mass spectrometry (LC/MS)^22^. Accumulation of NRE glycans in MPS disorders is usually quite dramatic in comparison to overall HS or glycosaminoglycan (GAG) accumulation because the biomarker is typically present at very low levels in normal tissues, whereas all cells make HS. Quantification of total GAG (as uronic acid) and the NRE biomarker characteristic of MPS IIIA (*N*-sulfoglucosamine) from whole brains of wild type, *Sgsh*^+/*h*^ and *Sgsh*^*h/h*^ mice at birth and postnatal days 8, 10, 14, 37, and 58–60 showed that GAG accumulation in the brain was apparent at birth (Fig. 3A), with striking accumulation of the diagnostic NRE biomarker at all time points (Fig. 3B). In mice, brain mass increases as cells are added during the first several postnatal weeks, a process that did not differ significantly between genotypes at any age (Fig. S3A). The extent of accumulation of NRE biomarker, when normalized to brain weight, did not change significantly over the 8-week observation period.

Disaccharide analysis also was performed using LC/MS, which provides compositional data as well as an independent means for assessing total HS content^23^. HS was significantly elevated in cerebral cortical homogenates prepared from *Sgsh*^*h/h*^ mice and increased modestly from 14 days to 58–60 days (Fig. 3C), as did NRE levels (Fig. 3D). NRE levels were elevated to a greater extent in cortical samples compared to whole brain (11 ± 2 (14d) and 16 ± 4 (58d) nmol NRE/g tissue wet weight in cortical samples versus 5 ± 1 (14d) and 7 ± 1 (58d) nmol NRE/g tissue wet weight in whole brain). The NRE biomarker was elevated to a similar extent at 14 days of age in the cerebral cortex of a novel MPS IIIA mouse strain bearing a null allele for *Sgsh (Sgsh*^−/−^) (see methods; compare Fig. 3E and B). Based on disaccharide analysis, the degree of 2-*O*-sulfation increased over time in *Sgsh*^*h/h*^ mice (Fig. S3B), with corresponding changes in 2-*O*-sulfated disaccharides at 58 days (Fig. S3C). In contrast, no changes in chondroitin/dermatan sulfate (CS/DS) levels or disaccharide composition were observed between genotypes (Fig. S3D–F). These results show that the developing postnatal cerebral cortex is heavily burdened with a pathological form of HS having an altered composition and characteristic NRE, which we refer to as “pathological HS”. Importantly, HS accumulates in the absence of secondary pathology and precedes synaptic dysfunction.

### Pathological heparan sulfate alters cell surface AMPA GluA2 subunits

**Figure 4:**
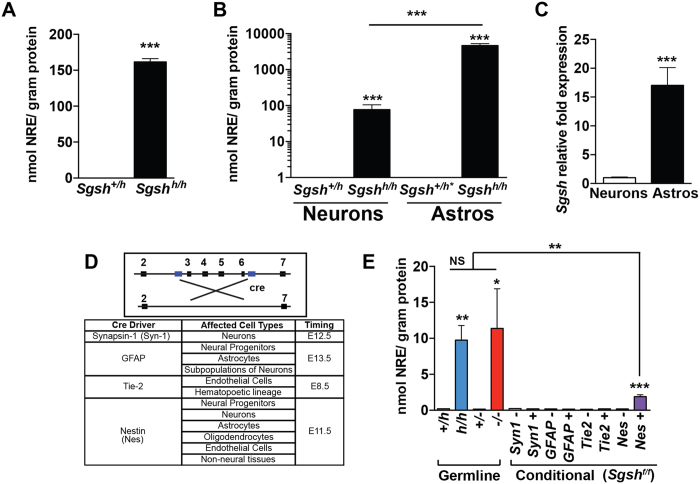
Contributions of neurons and astrocytes to HS accumulation in the cerebral cortex. (**A**) NRE levels in cell lysates prepared from primary mature cortical neural cultures containing a mixture of neurons and glia isolated from unaffected (*Sgsh*^+/h*^) and hypomorphic MPS IIIA (*Sgsh*^*h/h*^) mice. N = 3 individual animals per genotype spanning two litters. (**B**) NRE levels in intracellular preparations from mature primary cortical cultures of enriched neurons or astrocytes from unaffected and hypomorphic MPS IIIA mice. Note that differences are presented using a logarithmic scale. N = 3 individual animals per genotype spanning two litters. Cell preparations of neurons and astrocytes were obtained from different animals. (**C**) Relative *Sgsh* transcript expression by quantitative RT-PCR in wild type primary cultures enriched for neurons or astrocytes. N = 3–4 individual animals spanning two litters. (**D**) Genomic arrangement of conditional *Sgsh* allele and Cre-recombinase drivers. Table shows the affected cell types and developmental stage when cre recombinase is expressed for each strain. (**E**) NRE levels in the cerebral cortex from mice at 182 days of age. N = 3 animals per genotype.

To determine the cellular origin of HS bearing the NRE biomarker, primary cultures of highly enriched neurons or astrocytes (>95% purity) were prepared. Dramatic accumulation of NRE biomarker was present in cell lysates prepared from mixed primary cultures containing neurons and glia isolated from the cerebral cortex of newborn *Sgsh*^*h/h*^ mice (Fig. 4A). Most of this material derives from astrocytes based on 60-fold more NRE in enriched astrocyte preparations compared to isolated neurons (Fig. 4B). Evaluation of *Sgsh* transcript levels in available RNAseq datasets showed significantly higher expression of *Sgsh* transcripts in astrocytes than neurons, an observation that we confirmed in the astrocyte and neuronal cell cultures by qPCR (Fig. 4C)^24^. Several HS-degrading lysosomal hydrolases and genes that regulate lysosomal biogenesis, including transcription factor EB (*TCFEB*) also were enriched in astrocytes compared to neurons (Table S1).

Synapse formation has both a cell autonomous neuronal component and non-cell autonomous glial component. We attempted to measure the contribution of different cell types to disease pathogenesis *in vivo* by selective deletion of a “floxed” allele of *Sgsh (Sgsh*^*f/f*^) in astrocytes and other cell types found in the brain. Crossbreeding *Sgsh*^*f/f*^ mice to strains carrying different cell-specific Cre drivers (Fig. 4D) resulted in extensive recombination of the floxed *Sgsh* allele based on PCR analysis of DNA derived from cerebral cortex (Fig. S4A,B). However, inactivation of *Sgsh* in neurons (*Sgsh*^*f/f*^*Syn1-cre*^+^), astrocytes (*Sgsh*^*f/f*^*GFAP-cre*^+^) or endothelia/myeloid cells (*Sgsh*^*f/f*^*Tie2-cre*^+^) did not result in NRE biomarker accumulation in the cerebral cortex (Fig. 4E), most likely due to cross correction by secreted sulfamidase^25^. Consistent with this hypothesis, *Sgsh*^f/f^*Nes-cre*^+^ animals, which results in *Sgsh* inactivation in the majority of cells in the brain, accumulated NRE biomarker (Fig. 4E) and LAMP-1 immunoreactive lysosomes, albeit to a lesser extent than *Sgsh*^*h/h*^ (Fig. 2A) and *Sgsh*^−/−^ mice (Fig. S4C).

**Figure 5:**
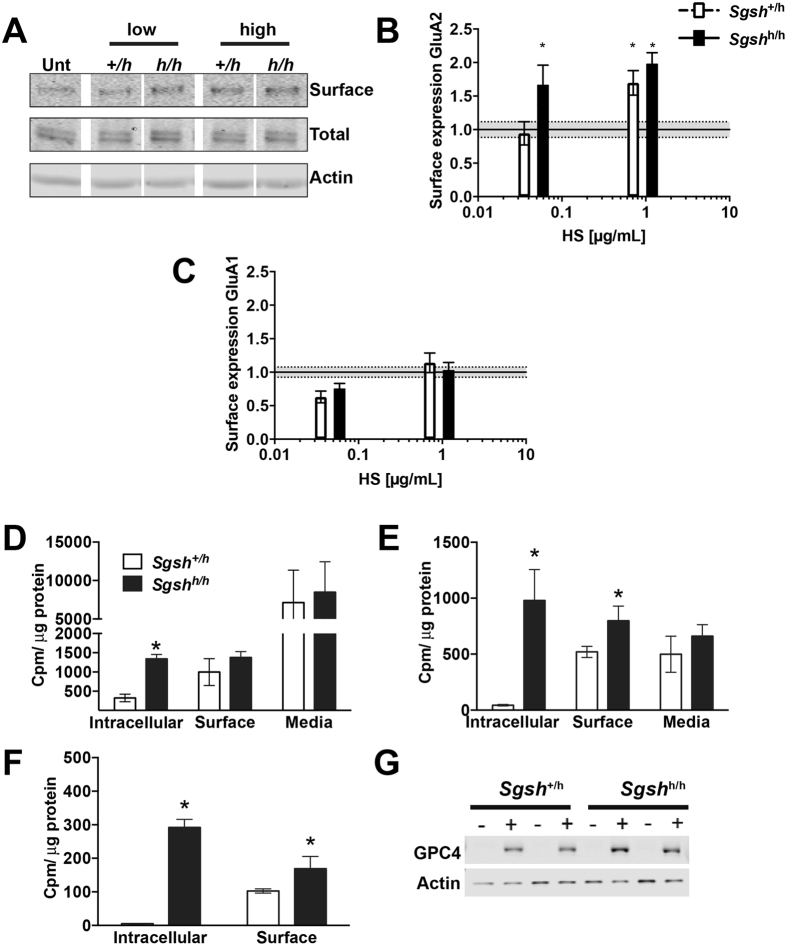
HS alters AMPA receptor subunit GluA2 recruitment to the cell surface. (**A**–**C**) Mature wildtype rat cortical neurons were treated for 72 hours with different brain equivalents of HS purified from the cerebral cortex of unaffected and hypomorphic MPS IIIA mice. Note that the mutant brain contains twice the amount of HS as the unaffected mice. (**A**) Western blots for GluA2 AMPA receptor subunit shows cell surface GluA2 levels are enhanced following addition of HS, while total GluA2 levels are unchanged. (**B**) Quantification of surface GluA2 levels normalized to total GluA2 from panel A. Mean ± SEM. (**C**) Quantification of surface GluA1 levels normalized to total GluA1 for the same samples in (**B**). Mean ± SEM. N = 3–6 wells per concentration from N = 3 experiments. (**B**,**C**) Horizontal line with grey shading shows control levels in untreated samples as mean ± SEM used for statistical comparison. (**D**) Purified GAGs from intracellular, cell surface, and media fractions from primary astrocyte cultures from unaffected (*Sgsh*^+/*h*^) and hypomorphic MPS IIIA (*Sgsh*^*h/h*^) mice following a 48-hour radiolabeling with ^35^SO_4_. (**E**) Purified GAGs from corresponding fractions following a 48-hour chase. (**F**) Purified HS from the fractions shown in panel E. Mean ± SD. N = 2 animals per genotype, 2 wells per animal. Results were confirmed on a separate cohort of N = 2 animals per genotype. (**G**) Western blot for glypican 4 in cell lysates of cultured primary astrocytes. Buffer control (−), heparin lyase treated (+).

Although we were unable to assess the contribution of astrocyte accumulation to the neurodevelopmental phenotype *in vivo*, we demonstrated that pathological HS that accumulates in the cerebral cortex affects the surface expression of AMPA receptors in the postsynaptic membrane of rat cortical neurons cultured *in vitro*. HS chains from the cerebral cortex of *Sgsh*^+/*h*^ or *Sgsh*^*h/h*^ mice, which mostly derives from resident astrocytes, were purified and added to cultures of mature wild type rat cortical neurons in equal “brain equivalents,” which mirrors the two-fold increase in overall brain HS in the mutant. Plasma membrane AMPA receptor subunit levels were assessed by cell surface biotinylation followed by precipitation with streptavidin beads and immunoblotting for AMPA receptor GluA2 or GluA1 subunits. The addition of HS from either the mutant or the unaffected animals did not affect the total level of GluA2 subunits (Fig. 5A), but plasma membrane associated GluA2 subunits were increased when HS from the mutant was added or when higher concentrations of HS were added (Fig. 5B). Note that the horizontal line with grey shading shows control levels of GluA receptor subunits in untreated samples. The addition of HS chains did not alter GluA1 levels, suggesting the elevated HS expressed in the mutant has a specific effect on AMPA receptor GluA2 subunits (Fig. 5C).

The high level of HS accumulation in astrocytes and the predominance of astrocytes in the brain suggest that these cells are the primary source of HS. Analysis of HS distribution in astrocytes by radiolabeling with ^35^SO_4_ showed that cell surface and secreted GAG levels did not differ in unaffected and mutant cells, but accumulation of intracellular GAG was obvious in the mutant (Fig. 5D). In a label-chase experiment, which tracks the turnover of metabolically labeled HS, both intracellular and cell surface GAG was increased in *Sgsh*^*h/h*^ astrocytes (Fig. 5E). Purification of HS from these samples confirmed elevation of HS at the cell surface (Fig. 5F). Glypican 4 levels were also modestly elevated in astrocyte lysates derived from *Sgsh*^*h/h*^ mice, although the difference did not reach significance (*Sgsh*^+/*h*^ 1.0 ± 0.37, *Sgsh*^*h/h*^ 1.36 ± 0.56) (Fig. 5G). These findings show HS supplementation is sufficient to increase GluA2 levels and shows a dependence on concentration and that astrocytes may be the primary source of HS that contributes to alterations in post-synaptic function.

## Discussion

In this report, we show that excitatory postsynaptic structure and function is abnormal in the developing somatosensory cortex in MPS IIIA mice, that HS accumulation is present at birth and remains at an elevated steady-state level in the developing postnatal brain, and that HS accumulation occurs prior to astrocytosis and secondary storage of ganglioside GM3. Furthermore, we show that HS accumulation mostly derives from astrocytes and that exogenous HS is sufficient to alter the level of AMPA receptor, GluA2, on the cell surface. The reduced excitatory synaptic strength observed in 21 day-old MPS IIIA mice coincides with the appearance of behavioral changes that have been published by other research groups^18^, suggesting a potential causal relationship. Together these findings provide the first evidence that neurodevelopmental abnormalities contribute to disease pathogenesis in MPS III.

To our knowledge, previous studies have not addressed whether pathological HS accumulates early in brain development and prior to histological hallmarks of neurodegeneration. In part, this lack of information reflects a technological limitation for assessing HS (and other GAGs) in small samples, a problem solved using LC/MS assessment of the MPS IIIA NRE biomarker, *N*-sulfoglucosamine. This method has a wide dynamic range, can detect storage in as little as 10^4^ cells or ≤1 mg wet weight of tissue, and has a signal (mutant) to noise (wildtype) ratio that cannot be achieved using methods that simply measure total GAG by dye binding or chemical methods. Using LC/MS NRE analysis, we show that storage of pathological HS occurs in the brain at birth and did not change over the first 8 postnatal weeks when normalized to brain weight, potentially explaining why the expected increase in NRE is not observed over this period. We also show that HS accumulates differentially in isolated cortical neurons and astrocytes. Other secondary markers of disease, including GM3^26^, CS/DS^27^, 2-*O*-sulfated HS derived disaccharides^15^, and astrocytosis are not present in the developing MPS IIIA brain, and should be considered as hallmarks of the later neurodegenerative phase of the disease. The delay in primary and secondary pathology likely reflects the progressive nature of neurological deterioration in the aging MPS IIIA brain.

Studies to evaluate alterations in synaptic function were performed primarily in the somatosensory cortex, which is a region of the brain involved in processing sensory information. A striking increase in the number of PSD-95 positive puncta was observed at 21 days of age prior to astrocytosis and ganglioside accumulation. In the normal brain, PSD-95 localizes to dendritic spine heads. However, the number of dendritic spines on pyramidal neurons in layer II/III did not differ from normal littermates and no change in mEPSC inter-event interval was observed. Together these data indicate that the enhancement in PSD-95 puncta does not reflect an increase in synapse number. Given the degree of HS accumulation in the 21-day old mouse brain, the absence of substantial alterations in synapse number is surprising and suggests that the establishment of the appropriate number of synaptic connections can occur even in the presence of lysosomal storage.

We observed a modest but consistent reduction in mEPSC amplitude. Reduced mEPSC amplitude was unexpectedly accompanied by enhanced dendritic spine width, suggesting that AMPA receptor function was reduced. Free HS chains are sufficient to alter AMPA receptor subunit levels at the cell surface based on the impact of adding HS isolated from the cerebral cortex to rat cortical neurons. The elevated level of glypican 4 and cell surface HS in MPS IIIA astrocytes support that astrocyte HS may be the primary source of HS that drives changes in postsynaptic function in MPS IIIA. Allen *et al*. recently showed that the addition of HS proteoglycans, glypicans 4 or 6, to retinal ganglion cells increases mEPSC amplitude and promotes the formation of excitatory synapses, whereas deletion of glypican 4 reduces mEPSC amplitude^7^. Irie *et al*. reported that deletion of the HS co-polymerase *Ext1* in post-mitotic neurons led to a reduction in plasma membrane levels of glutamate AMPA receptors, specifically GluA2, and a modest but significant reduction in mEPSC amplitude^9^. The sensitivity of this receptor system to both elevated and reduced HS levels is consistent with the bell shaped response curves reported by Allen and colleagues^7^. Glypican 4 may be just one of several HSPGs that accumulate in MPS III, based on the observation that MPS IIIB mice have elevated levels of glypican 1 in the medial entorhinal cortex^28^.

Sambri and colleagues recently reported lysosomal impairment in 10-month-old MPS IIIA mice causes a loss of presynaptic function in the hippocampus by inhibiting synaptic vesicle trafficking^29^. Thus, we suggest that different forms of synaptic impairment likely cause changes in neurological function in the developing and aged MPSIIIA brain, consistent with progressive neurological deterioration associated with this disorder. Future studies to develop therapeutic treatments will benefit from this enhanced understanding of the disease process.

Only limited information is available on the contribution of different neural cell types to disease pathology in MPS III^30^,^31^. Towards this goal, we examined a new mouse model carrying a conditional allele of *Sgsh*, driving deletion of *Sgsh* in specific cell types by intercrossing *Sgsh*^f/f^ mice to animals expressing Cre recombinase in different neural cell types, including neurons, endothelial cells, and astrocytes. The lack of NRE biomarker accumulation in conditional knockout mice shows that cross-correction by secreted sulfamidase apparently occurs across different cell types in the brain. Lower, but significant levels of biomarker were observed in *Sgsh*^*f/f*^*Nestin-Cre* mice, suggesting that a non-neuroepithelial cell type (microglia, meningeal fibroblasts, and ependymal cells) may also participate in cross-correction in the brain.

In summary, we show for the first time that MPS IIIA has a neurodevelopmental component most likely induced by accumulation of pathological forms of HS. Further studies are warranted to explore the relationship of HS content and composition in synaptic biology not only in MPS disorders, but also in other neurodevelopmental and neurodegenerative disorders that have shared characteristics with MPS III^32^.

## Materials and Methods

### Mouse models

Hypomorphic MPS IIIA mice were purchased from Jackson Laboratory (*B6.Cg-Sgshmps3a/PstJ*)^17^. These mice carry a homozygous missense mutation in the *Sgsh* gene and exhibit <5% residual activity of sulfamidase. A mouse model of MPS IIIA bearing a conditional “floxed” allele of *Sgsh* was obtained from the Knockout Mouse Project Repository (*Sgsh*^tm1a(KOMP)Wtsi^). The *Sgsh* “knockout first” allele was converted to a conditional allele via flippase-mediated recombination, which exposed two lox-P sequences flanking exons 3 through 6 of *Sgsh*. Subsequent crossing to animals bearing germ-line expression of bacterial Cre recombinase eliminated these 4 exons and induced a frame-shift mutation at the junction between exons 2 and 7 and a premature stop codon, thus abolishing enzyme function and/or synthesis of the enzyme. *Sgsh*^f/f^ mice were crossed to *Nestin-cre* mice (Jackson Laboratory, *Tg(Nes-cre)2Wme*), *Synapsin1-cre* (Jackson Laboratory, *B6.Cg-Tg(Syn1-cre)671Jxm/J*), and a *GFAP-cre* line (generated by the Messing Laboratory and provided by Don Cleveland, UCSD^33^. To generate a mouse line bearing a null allele of *Sgsh, Sgsh*^*f*/+^ mice were crossed to *EIIA-cre* mice purchased from Jackson Laboratory (*Bg.FVB-Tg(EIIa-cre)C5379Lmgd/L*). Female offspring of genotype *Sgsh*^f/+^*EIIA-cre*+ were crossed to wildtype C57BL/6 h mice to obtain germline transmission of the null *Sgsh* allele and to remove the cre transgene. Heterozygous offspring were bred to produce wild type, *Sgsh*^+/−^ and *Sgsh*^−/−^ offspring. Supplemental Table S2 contains primer sequences for genotyping.

Plug discovery was counted as embryonic day 0 (E0) and the date of birth was defined as postnatal day 0 (P0). Male and female mutant mice were analyzed and gender was carefully monitored in all experiments. Unaffected littermate animals of the same gender were analyzed to control for inter-litter and male/female variation. In all experiments gender did not systematically vary. Data collected in experiments employing different experimental techniques was generated using separate cohorts of mice. Animals were housed and bred in vivaria approved by the Association for Assessment and Accreditation of Laboratory Animal Care located in the School of Medicine, UCSD. All experiments were performed in accordance with relevant guidelines and regulations following standards and procedures approved by the UCSD Institutional Animal Care and Use Committee.

### Slice Electrophysiology

Acute hippocampal and cortical slices were prepared from 21–22 day old *Sgsh*^+/*h*^ and *Sgsh*^*h/h*^ mice with the experimenter blinded to genotype. Mice were deeply anesthetized with isofluorane prior to decapitation and brains were incubated at 4 °C in modified artificial cerebrospinal fluid (ACSF) containing sucrose (83 mM NaCl, 2.5 mM KCl, 1 mM NaH_2_PO_4_, 26.2 mM NaHCO_3_, 22 mM glucose, 72 mM sucrose, 0.5 mM CaCl_2_, and 3.3 mM MgSO_4_). Coronal slices were prepared (350 μm) using a Leica VT1200 vibratome and recovered in standard ACSF (119 mM NaCl, 5 mM KCl, 1 mM NaH_2_PO_4_, 26 mM NaHCO_3_, 11 mM glucose, 2 mM CaCl_2_, and 1 mM MgSO_4_). The slices were incubated at 34 °C for 30 min and at room temperature prior to recordings. Slices were transferred to a submerged recording chamber and perfused with 30 °C oxygenated ACSF (containing 0.1 mM picrotoxin). For miniature excitatory postsynaptic current (mEPSC) recordings, 1 μM tetrodotoxin (TTX) was included in the circulating ACSF.

For mEPSC recordings, neurons displaying pyramidal-like morphology in layer II/III of primary somatosensory cortex were whole-cell voltage-clamped at −70 mV with glass pipettes of 2.5–3.5 MΩ resistance filled with a cesium-based internal solution (10 mM CsCl, 105 mM CsCH_3_SO_3_, 0.5 mM ATP, 0.3 mM GTP, 10 mM HEPES, 5 mM glucose, 2 mM MgCl_2_, and 1 mM EGTA, pH 7.2). mEPSC amplitude and inter-event interval were analyzed from 80–150 events per cell using Clampfit 10.3 and differences were determined using unpaired t-test in GraphPad Prism.

### Immunohistochemistry

Mice of various ages were heavily anesthetized using a mixture of ketamine/xylazine and transcardially perfused dPBS followed by 4% phosphate-buffered paraformaldehyde (PB-PFA). Tissues were post-fixed overnight at 4 °C and cryo-protected by sinking in 30% PB-sucrose at 4 °C and cut on a cryostat. Coronal sections (40 μm) were collected and stained as free-floating sections. Primary antibodies used were LAMP-1 (1D4B, Developmental Studies Hybridoma Bank, University of Iowa), glial fibrillary acidic protein (GFAP) (Sigma, G3893), and ganglioside GM3 (Cosmo Bio Co., NBT-M101). Confocal images were collected on a Nikon Ti microscope equipped with the Nikon A1R confocal system using a high NA 63X oil, 10X, or 20X objectives. The ND acquisition tool was used to acquire Z stacks in three channels. Quantification of LAMP-1 vesicle size was performed on high magnification max projection Z-stacks that span 4 μm in depth using the puncta analyzer Image J plugin. Quantification of area stained by GFAP and GM3 was performed on images set to the same threshold and area quantification was performed using Image J.

For staining of PSD-95 in tissue sections, PBS perfused whole brains were dissected and cut down the midline. The right hemisphere was flash frozen in OCT in a bath of isopentane that had been pre-cooled in liquid nitrogen. Tissue was transferred to dry ice and frozen at −80 °C for a minimum of one day. Sagittal sections (12 μm) were cut using a cryostat and mounted directly onto glass slides. Glass slides were fixed for 8 min at −20 °C in methanol, and then washed and fixed for 4 min in 4% PB-PFA at room temperature. Sections were stained with an anti-PSD95 antibody (LifeTech 51-6900) as previously described^34^.

Species-specific Alexa Fluor-conjugated secondary antibodies were used to visualize primary antibody staining. Confocal images were collected on a Nikon Ti microscope equipped with the Nikon A1R confocal system using a high NA 63X oil objective. The ND acquisition tool was used to acquire Z stacks in three channels. Quantification of PSD95 was performed using the puncta analyzer ImageJ plugin on 5 μm max projection confocal images constructed from z-stacks. Data from *Sgsh*^*h/h*^ sections were normalized to unaffected littermates that were sectioned and stained at the same time. N = 5–6 animals per genotype, 2 sections per animal, 2 images per section, 2 regions of interest per image. Golgi impregnation and dendritic spine analysis and quantification were performed exactly as described^35^.

### Western blot analysis

SDS-PAGE gels were cast manually. After electrophoresis of samples, bands were transferred to either nitrocellulose or PVDF membrane. Western blotting was performed as previously described^36^.

### Glycosaminoglycan Purification

Mice were heavily anesthetized using a ketamine/xylazine mixture, and transcardial perfusion with Dulbecco’s PBS (dPBS) was performed. Whole brains were removed and dissected into anatomical sub regions, when specified. Organs were homogenized in ice-cold buffer containing 50 mM sodium acetate (pH 6.0) and 0.2 M sodium chloride. The tissue was dissociated with a Polytron homogenizer, and the resulting homogenates were digested overnight at 37 °C with 0.1 mg/ml Pronase (type XIV from *Streptomyces griseus*, Sigma Aldrich) containing 0.1% Triton X-100. Tissue homogenates were filtered through 0.45 μm syringe filters or centrifuged at 10,000× g for 10 min to remove insoluble debris. Glycosaminoglycans were purified from the supernates by anion exchange chromatography as described previously^37^.

### Non-reducing end and glycosaminoglycan analysis

The disaccharides resulting from enzymatic depolymerization were dried and tagged with [^12^C_6_] aniline as described^23^. Each sample was mixed with a known amount of [^13^C_6_] aniline tagged standards, including *N*-sulfoglucosamine, the primary saturated non-reducing end biomarker for MPS IIIA (Carbosynth). Samples were analyzed by liquid chromatography-mass spectrometry (LC/MS) using an LTQ Orbitrap Discovery electrospray ionization mass spectrometer (Thermo Scientific) as described^22^. Internal disaccharides and the non-reducing end monosaccharide were quantified manually based on their unique mass signatures relative to internal standards and normalized by either tissue wet weight or protein concentration, determined by BCA assay (Pierce). Data is presented using a disaccharide structural code^38^.

### Primary Neuron and Astrocyte Isolation

Mixed cortical cultures were prepared from single newborn MPS IIIA pups as described^39^. Cells were cultured for 21 days prior to NRE and morphological analyses. Enriched cultures of primary cortical neurons were prepared from single mouse embryos (E15.5) as described^40^. To select for post-mitotic neurons, 5 μM cytosine arabinoside (AraC) was added at 1 day and removed with a medium change at 3 days.

Astrocyte cultures were obtained from the cortex of single newborn mice as described^41^. For NRE analysis, cells cultured to maturation (18–21 days) were treated with 0.05% trypsin-EDTA to remove cell surface HSPGs. Intact cells containing intracellular storage material were then collected by centrifugation. RNA was isolated from enriched cultures of wild type or heterozygous neurons and astrocytes and *Sgsh* expression was assessed by qRT-PCR (primer sequences, Table S2).

### Data quantification and statistical analyses

In all cases a researcher blinded to genotype performed image quantification. Statistical analyses were performed using GraphPad Prism software Synaptic analyses are presented as average values ± SEM to take into account the large number of samples and size of datasets, whereas other data is presented as average values ± SD. *p < 0.05, **p < 0.01, ***p < 0.001.

Additional details for experimental procedures including mouse breeding scheme, immunohistochemistry, glycosaminoglycan purifications, primary cultures for neuron and astrocyte enrichment, slice electrophysiology for field recordings, Western blot analysis, evaluation of surface AMPA receptor levels, and metabolic label/chase experiments can be found in Supplementary Information under Materials and Methods.

## Additional Information

**How to cite this article**: Dwyer, C. A. *et al*. Neurodevelopmental Changes in Excitatory Synaptic Structure and Function in the Cerebral Cortex of Sanfilippo Syndrome IIIA Mice. *Sci. Rep.* **7**, 46576; doi: 10.1038/srep46576 (2017).

**Publisher's note:** Springer Nature remains neutral with regard to jurisdictional claims in published maps and institutional affiliations.

## Supplementary Material

Supplementary Information
