# Supplementary material for: Neurodevelopmental Changes in Excitatory Synaptic Structure and Function in the Cerebral Cortex of Sanfilippo Syndrome IIIA Mice
Source: Sci Rep. 2017 Apr 18;7:46576. doi: 10.1038/srep46576 (PMC5394534; doi:10.1038/srep46576)
Supplement: Supplementary Information [file srep46576-s1.pdf]

Supplementary Information

Neurodevelopmental Changes in Excitatory Synaptic Structure and Function in the Cerebral Cortex of Sanfilippo Syndrome IIIA Mice

Chrissa A. Dwyer[1*], Samantha L. Scudder[2], Ying Lin[1], Lara E. Dozier[2], Dustin Phan[1], Nicola J. Allen[3], Gentry N. Patrick[2] and Jeffrey D. Esko[1*]

[1] Department of Cellular and Molecular Medicine, Glycobiology Research and Training Center, University of California, San Diego, La Jolla, CA, 92093-0687, USA.
[2] Section of Neurobiology, Division of Biological Sciences, University of California, San Diego, La Jolla, CA 92093-0347 USA.
[3] Salk Institute for Biological Studies, Molecular Neurobiology Laboratory, La Jolla, CA 92037 USA.

*Corresponding authors:
Jeffrey D. Esko, Department of Cellular and Molecular Medicine, University of California, San Diego, La Jolla, CA 92093-0687, Ph: 858/822-1100, FAX: 858/534-5611, jesko@ucsd.edu

Chrissa A. Dwyer, Department of Cellular and Molecular Medicine, University of California, San Diego, La Jolla, CA 92093-0687, Ph: 858/822-1102, FAX: 858/534-5611, cdwyer@ucsd.edu

Supplementary Materials and Methods

Mouse breeding scheme. In all cases hemizygous expression of cre recombinase was maintained by breeding male mice carrying the cre transgene. Sgsh female dams heterozygous for hypomorphic and null alleles were used for breeding to prevent phenotypic drift.

Immunohistochemistry. For staining with LAMP-1, GFAP, and GM3, free-floating sections were blocked in 3% BSA in 1XPBS containing 0.1% Tween-20 (PBST) and 1% TritonX-100 for 45 min at room temperature. Primary antibodies were diluted in 3% BSA in 1XPBST containing 0.5% TritonX-100 and rotated at 4°C for 18-20 hr. Sequential staining was performed for GFAP, followed by GM3. Ig subclass-specific secondary antibodies were used.

For staining of brain sections with PSD-95, fixed slides were blocked for 45 min with 10% goat serum in PBS containing 0.2% Triton X-100 at room temperature and stained overnight at 4°C with anti-PSD95 antibody (LifeTech 51-6900) diluted in 3% BSA/1XPBST.

Preparation of purified glycosaminoglycans and LC/MS analysis. Purified GAG samples were treated with 20,000 U/ml DNAse I (bovine pancreas, Sigma, D-4263) for 2 hr at 37°C. β-elimination was then performed by adjusting samples to 0.4 M sodium hydroxide and incubation overnight at 4°C. Samples were then neutralized to pH 7.0 with acetic acid, diluted 20-fold and re-purified by anion exchange chromatography. For disaccharide analysis of chondroitin/dermatan sulfate (CS/DS), de-salted GAG samples were depolymerized with 20 mU of chondroitinase ABC (Seikagaku). For disaccharide analysis of HS, de-salted GAG samples were depolymerized using 2 mU each of heparin lyases I, II and III. The liberated disaccharides were processed over a strong protonated cation-exchange resin (Dowex 50). Samples were neutralized with 0.1 M ammonium hydroxide, frozen and lyophilized.

Mixed glial cultures and astrocyte enrichment. Mixed glial cultures were obtained by mechanical dissociation. Confluent astrocyte monolayers were observed at 10 days. Cells were then shaken for 8 hr at 250 rpm on an orbital shaker to remove contaminating microglia and oligodendrocyte precursor cells. Astrocytes were then passaged into 6-well plates and changed to serum-free growth medium (Neurobasal, 3% B27, 1X Glutamax, 1X Pen/Strep). Astrocytes were cultured for an additional 12 days, with media changes every 3 days, prior to being used in experiments.

Slice electrophysiology and field recordings. For field excitatory postsynaptic potential (fEPSP) experiments, a cluster stimulating electrode (FHC) was placed in the stratum radiatum of the CA1 region of the hippocampus and current was injected using an ISO-Flex stimulus isolator (A.M.P.I.) triggered by a Clampex 10.3 (Molecular Devices) protocol. Recording electrodes were generated from thin-walled capillary tubing (Warner Instruments) using a horizontal pipette puller (DMZ Zeitz Puller), resulting in a resistance of 2-3 MΩ, and were filled with ACSF. The recording electrode was placed 150-200 µm away from the stimulating electrode in the stratum radiatum, along a pathway parallel to the CA1 pyramidal layer. fEPSPs were evoked using 100 µsec pulses. Input-output relationships were determined by recording fEPSPs at a variety of stimulus intensities ranging from 30 µA to 130 µA and averaging five traces per intensity. Paired pulse facilitation was examined by generating pulses at variable separations (400, 200, 150, 100, and 50 msec) and taking the ratio of the second fEPSP amplitude to the first, with three recordings averaged for each separation. Field responses were recorded using an Axopatch 200B amplifier (Molecular Devices) and digitized using a Digidata 1322A digitizer (Molecular Devices), and signals were acquired using Clampex 10.3.

Western blot analysis. An anti-PSD95 antibody (LifeTech 51-6900) and anti-glypican 4 (ProteinTech 13048-1-AP) were used. Digestions with heparin lyase enzymes (I-III) were performed by incubating cell lysate in 1X digestion buffer (50mM Tris pH 8.0, 50mM NaCl, 2.5mM $MgCl_2$, 0.5mM $CaCl_2$, 2.5mM KCl, EDTA-free protease inhibitor tablets) with 1mU of each enzyme for 6 hr at 37°C. Quantification of Western blots was performed by measuring band pixel intensity in Image J and normalizing values to β-actin band intensity.

Evaluation of surface AMPA receptor levels. Mixed cortical neuron cultures were prepared from newborn wildtype rats and cultured to maturation (21 days). Heparan sulfate was purified from the cerebral cortex of *Sgsh*$^{+/h}$ or *Sgsh*$^{h/h}$ mice. To recapitulate elevated HS levels in *Sgsh*$^{h/h}$ mice, volumes used to reconstitute lyophilized HS were normalized to wet weight of tissue samples used for the purification (referred to as “brain equivalents”). Equal brain equivalents of purified HS from respective genotypes were added to mature wild type rat cortical mixed neuron cultures (18 days) in 6 well plates containing 2mL of growth medium. Concentrations of HS added to each well were calculated by liquid chromatography/mass spectrometry based analysis of disaccharide content [1].

Cultures were incubated for 3 days without media changes and then processed for cell surface biotinylation. Plates were chilled on ice, washed 3X in ice cold PBS, and incubated in 125 µg of EZ link sulfo NHS-LC Biotin (Pierce) diluted in PBS for 15 min on ice. To remove unreacted biotin, cells were washed 3X in cold PBS and then lysed in 1X RIPA buffer containing protease inhibitors (Roche). Samples were solubilized by incubating at 4°C for 1 hr while rotating and then centrifuged at 16,000 x g for 15 min to pellet insoluble material. A fraction of the soluble material was retained as input (total). 0.1mg of total protein was processed for extraction of biotinylated surface proteins using streptavidin beads (Pierce). AMPA receptor subunits were analyzed by SDS-Page and Western blotting for antibodies against GluA2 (Millipore) and β-actin (Sigma). Optical densities of resulting bands were quantified by Image J. The amount of surface AMPA receptor subunits were normalized to input material (total levels).

Cell surface biotinylation of acute brain slices for detection of surface GluA1 and GluA2 levels in the primary somatosensory cortex was performed as described [2].

Turnover of $[^{35}S]$ Glycosaminoglycans. Primary astrocyte cultures were aged and radiolabeled for 48 hr by incubating cells in neurobasal medium supplemented with 3X B27, 1X glutamax, 200 µCi/ml $Na[^{35}S]O_4$ (PerkinElmer Life Science). To monitor turnover cells were chased for 48hr by rinsing cells twice in PBS and incubating in neurobasal medium supplemented with 3X B27, 1X glutamax, 1X Pen/Strep. Cells were washed in PBS and incubated in 0.05% trypsin-EDTA to lift the cells and release cell surface proteoglycans. Cell pellets were lysed in 0.1N NaOH and used to measure protein concentration by BCA assay. Media, surface and intracellular fractions were processed for glycosaminoglycan purification as described [3].

Supplementary Tables

Supplementary Table S1. Differences in cellular expression levels of genes encoding lysosomal biosynthetic proteins and GAG degrading enzymes. Data from RNAseq profiling of astrocyte and neuron transcriptomes, Zhang and colleagues [4]. FPKM, Fragments Per Kilobase of transcript per Million mapped reads; A, astrocytes; N, neurons.

Function	Gene symbol	Description	Raw FPKM		Normalized Fold Expression
			A	N	A:N
Lysosome Biosynthesis	**Tcfeb**	Transcription Factor EB (TFEB)	5.2	1.1	6.28
	**Lamp1**	Lysosomal-associated membrane protein 1	233.6	97.6	3.24
	**Lamp2**	Lysosomal-associated membrane protein 2	71.5	17.8	5.44
MPSI	**Idua**	Iduronidase, alpha-L-	5.1	1.3	5.17
MPSII	**Ids**	Iduronate 2-sulfatase	13.1	70.4	0.25
MPSIIIA	**Sgsh**	N-sulfoglucosamine sulfohydrolase	2.7	0.5	7.32
MPSIIIB	**Naglu**	N-acetylglucosaminidase, alpha	5.6	2.8	2.71
MBSIIIC	**Hgsnat**	Heparan-alpha-glucosaminide N-acetyltransferase	10.6	11.6	1.24
MPSIIID	**Gns**	Glucosamine (N-acetyl)-6-sulfatase	22.5	20.3	1.51
MPSIIIE	**Arsg**	Arylsulfatase G	2.3	2.7	1.13
MPSVI	**Arsb**	Arylsulfatase B	13.3	24.4	0.74
MSD	**Sumf1**	Sulfatase modifying factor 1	4.5	3.6	1.69

Supplementary Table S2. Primer Sequences

qRT-PCR		
**Gene**	**Forward Primer (5'-3')**	**Reverse Primer (5'-3')**
Sgsh	CAGCCTTATCTTCCGTAACGC	GCCCATACATGCCATTCTGAT
Ywhaz	CCTGCATGAAGTCTGTAACTGAG	GACCTACGGGCTCCTACAACA
18s	CGCCGCTAGAGGTGAAATTC	TTGGCAAATGCTTTCGCTC

Genotyping (*Sgsh*$^{f/f}$)			
**Primer**	**Forward Primer (5'-3')**	**Reverse Primer (5'-3')**	**Amplicon Size**
Floxed	1. GAGATGGCGCAACGCAATTAATG	2. AGGCAGATCTCTGTGAGTTTGAGACC	Floxed only (343bp)
Wildtype	3. TATGTTGTGTGAGCTGGTGAGGTGG	4. TGTAAAAGGGTTGGGGCTGTGC	Wildtype (186bp), Floxed (294bp)
Deleted	5. GGTGGAACACACCATACACG	6. GGATCCGATGCCATCTTCTA	Deleted (204bp)

Supplementary Figures

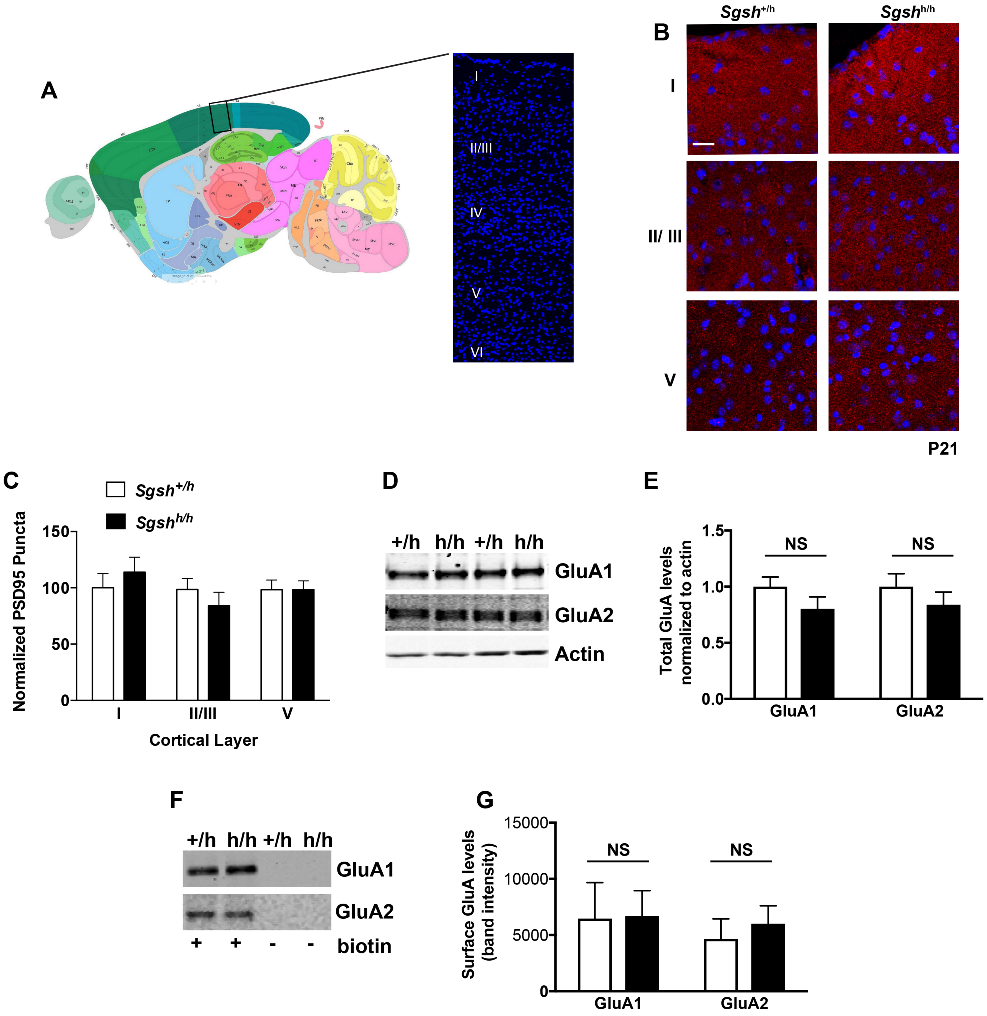

Figure S1. Characterization of excitatory postsynaptic alterations in the primary somatosensory cortex of postnatal MPS IIIA mice

A Diagram of sagittal mouse brain section showing region of the primary somatosensory cortex analyzed (boxed). Image credit: Allen Institute. © 2004 Allen Institute for Brain Science. Allen Mouse Brain Atlas. Available from: http://mouse.brain-map.org/static/atlas, sagittal atlas, image 11 of 21. Inset shows DAPI stained image of cortical layers analyzed. **B** Representative maximum intensity projection Z-stacks showing an enlarged area of the primary somatosensory cortex from 21d mice stained with PSD-95 (red) from selected insets presented in Fig. 1A. Different images were acquired in cortical layers I, II/III, and V. Scale, 50 μm. Nuclei are stained with DAPI (blue). **C** Quantification of PSD95 puncta in cortical layers I, II/III, and V of the primary somatosensory cortex of 14d unaffected (*Sgsh*$^{+/h}$) and hypomorphic MPSIIIA (*Sgsh*$^{h/h}$) mice. Mean ± SEM. N=3 animals per genotype. Results show PSD-95 enhancement is not detected at 14d. **D** Western blot for GluA1 and GluA2 levels in cortical homogenate from 21d unaffected and hypomorphic MPS IIIA mice. **E** Quantification of GluA1 and GluA2 levels from D. Band pixel intensities were measured in ImageJ and normalized to β-actin. **F** Western blot analysis for cell surface GluA1 and GluA2 in acute brain slices from 21d unaffected and hypomorphic MPS IIIA mice with and without biotinylation. **G** Quantitation of panel F. Mean ± SEM. N=6 animals per genotype, spanning 3 litters.

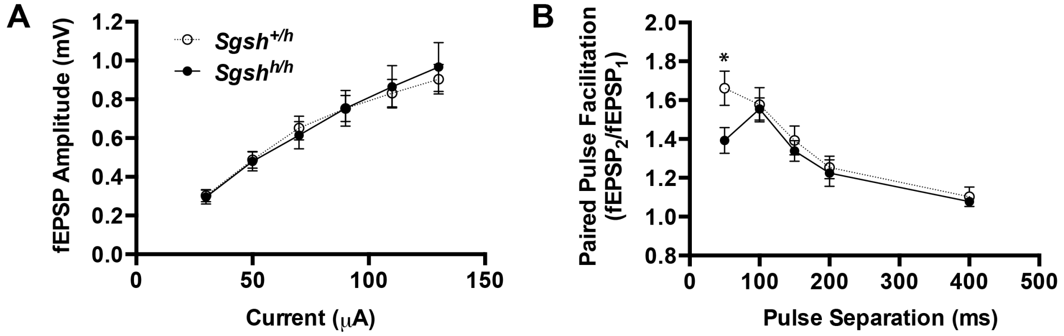

Figure S2. Normal hippocampal synaptic transmission in MPS IIIA mice
A Field EPSP amplitudes in response to increasing intensities of Schaffer Collateral stimulation (30, 50, 70, 90, 110, and 130 µA) are unaltered in acute hippocampal slices from 21-22 d hypomorphic MPS IIIA mice. Mean ± SEM. N= 3 mice per genotype, 7-5 slices total. **B** Ratio of response to a second Schaffer collateral stimulation to the first at varying pulse separations (50, 100, 150, 200, and 400 ms), with similar facilitation at short separations in both genotypes. N=3 mice per genotype, 6-7 slices total. Mean ± SEM.

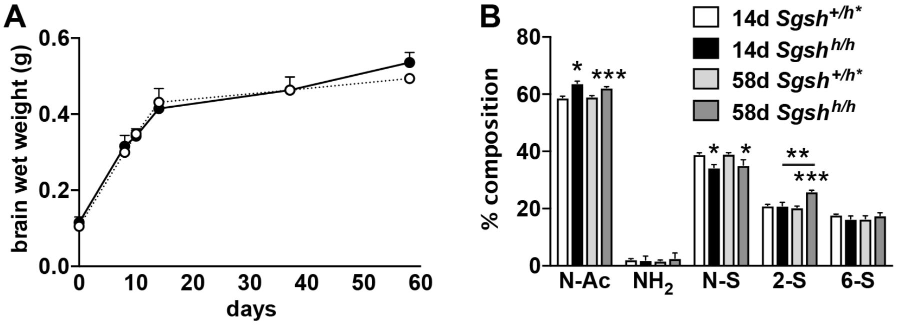

C

Heparan Sulfate

		D0A0	D0S0	D0A6	D2A0	D2H6	D0S6	D2S0	D2S6
		% of total disaccharides							
14d	$Sgsh^{h/+}$	51 ± 1	16.6 ± 0.1	6.4 ± 0.1	1.8 ± 0.1	1.20 ± 0.3	4.7 ± 0.1	12.7 ± 0.3	5.0 ± 0.3
	$Sgsh^{h/h}$	56 ± 1.2	13.5 ± 0.1	5.9 ± 0.3	1.7 ± 0.2	1.0 ± 1.5	2.9 ± 0.3	11.6 ± 0.2	6.3 ± 1.0
58d	$Sgsh^{h/+*}$	52 ± 0.5	16.2 ± 0.4	6.1 ± 0.1	1.2 ± 0.2	1.0 ± 0.4	4.9 ± 0.1	13.8 ± 0.5	4.3 ± 0.7
	$Sgsh^{h/h}$	56 ± 0.5	9.6 ± 0.2	5.0 ± 0.1	1.1 ± 0.1	1.7 ± 1.9	2.9 ± 0.2	15.0 ± 0.9	7.8 ± 0.9

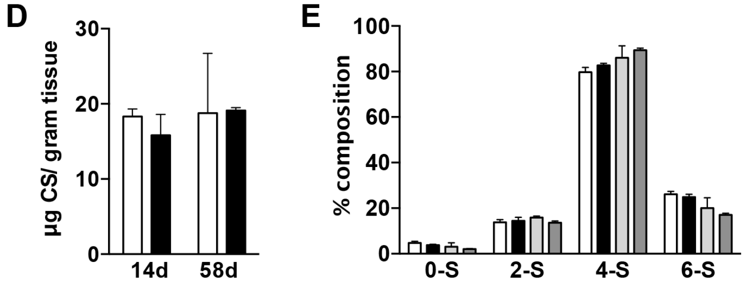

F

Chondroitin/ Dermatan Sulfate

		D0a0	D0a4/D2a0	D0a6	D2a4	D2a6	D0a10	D2a10
		% of total disaccharides						
14d	$Sgsh^{h/+}$	4.7 ± 0.6	63 ± 2	10 ± 2	6.0 ± 0.6	6.0 ± 0.4	9.0 ± 0.1	1.6 ± 0.2
	$Sgsh^{h/h}$	3.8 ± 0.4	64 ± 2	8 ± 1	6.9 ± 0.6	5.6 ± 0.7	9.5 ± 0.2	1.9 ± 0.4
58d	$Sgsh^{h/+*}$	3 ± 2	71 ± 5	3 ± 2	6 ± 1	8 ± 2	7.5 ± 1	1.8 ± 0.3
	$Sgsh^{h/h}$	2 ± 0.2	75 ± 1	2.3 ± 0.2	5.8 ± 0.5	6.3 ± 0.6	6.8 ± 0.1	1.6 ± 0.2

Figure S3. Postnatal brain mass and glycosaminoglycan disaccharide compositional analysis in MPS IIIA mice

A Whole brain wet weights from GAG and NRE measurements in Fig. 3A, B. Mean ± SD. **B** Non-sulfated, N-sulfated, 2-O-sulfated, and 6-O-sulfated disaccharides derived from cerebral cortex HS. Mean ± SD**. C** Cerebral cortex HS disaccharide quantification by LC/MS. Mean ± SD. **D** Cerebral cortex chondroitin/dermatan sulfate levels measured by LC/MS. Mean ± SD. **E** Cerebral cortex chondroitin/dermatan sulfate composition by LC/MS. Mean ± SD. **F** Cerebral cortex chondroitin/ dermatan sulfate disaccharide quantification by LC/MS. Mean ± SD. Unaffected wild type and heterozygous (*$Sgsh^{+/h*}$* open bars) and hypomorphic MPS IIIA (*$Sgsh^{h/h}$* closed bars), unless otherwise specified. Day of birth = 0d. N=3 animals per genotype per age. Standardized glycosaminoglycan disaccharide code is used [5].

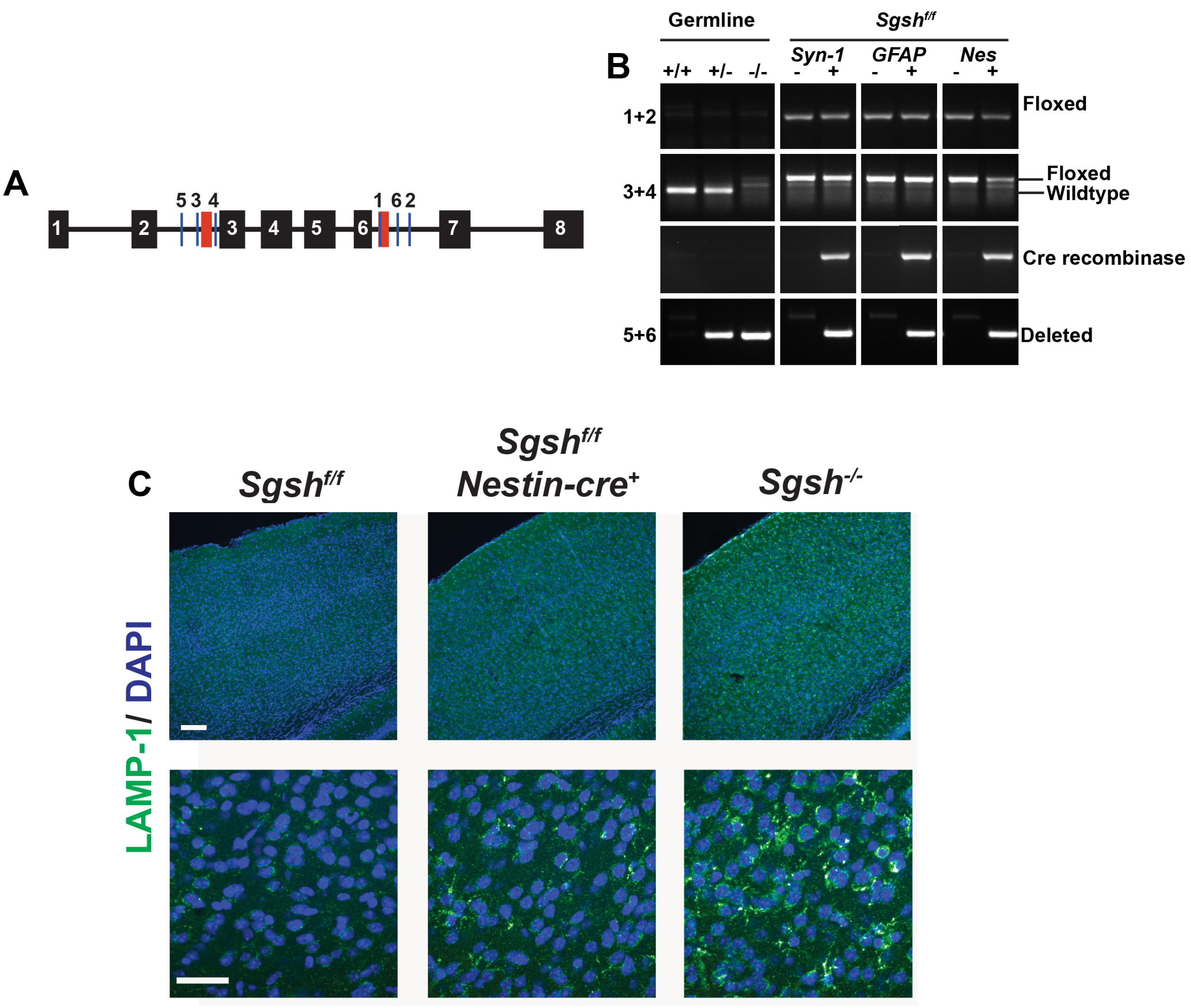

Figure S4. Deletion of *Sgsh* occurs in cortex of conditional mouse strains and lysosomal expansion occurs in the *Sgsh*$^{f/f}$*Nestin-cre*+ somatosensory cortex

A Location of conditional and null genotyping primers (blue lines). LoxP sites shown in red. **B** Gel electrophoresis showing PCR bands obtained for floxed, floxed/wildtype, cre recombinase, and deleted allele from genomic DNA isolated from the cerebral cortex of respective mouse strains. Band showing deleted allele arises from genomic recombination induced by cre activity. **C** Representative confocal images of the primary somatosensory cortex from unaffected (*Sgsh*$^{f/f}$), *Sgsh*$^{f/f}$*Nestin-cre*+, and germline Sgsh null (*Sgsh*$^{-/-}$) from 61-day-old mice stained with the lysosomal marker, LAMP-1 (green). Top panel show low magnification images, scale bar 100 µm. High magnification max projection Z-stacks from somatosensory cortical layer II/III, scale bar 50 µm. In all cases nuclei were stained with DAPI (blue). N=4 animals per genotype.

References

1 Lawrence, R. *et al.* Evolutionary differences in glycosaminoglycan fine structure detected by quantitative glycan reductive isotope labeling. *J Biol Chem* **283**, 33674-33684 (2008).

2 Holman, D. & Henley, J. M. A novel method for monitoring the cell surface expression of heteromeric protein complexes in dispersed neurons and acute hippocampal slices. *J Neurosci Methods* **160**, 302-308 (2007).

3 Esko, J. D. in *Current protocols in molecular biology* (eds F. Ausubel *et al.*) 17.12.11-17.12.19 (Greene Publishing and Wiley-Interscience, 1993).

4 Zhang, Y. *et al.* An RNA-sequencing transcriptome and splicing database of glia, neurons, and vascular cells of the cerebral cortex. *J Neurosci* **34**, 11929-11947 (2014).

5 Lawrence, R., Lu, H., Rosenberg, R. D., Esko, J. D. & Zhang, L. Disaccharide structure code for the easy representation of constituent oligosaccharides from glycosaminoglycans. *Nat Methods* **5**, 291-292 (2008).
